# Arabidopsis ADF5 Acts as a Downstream Target Gene of CBFs in Response to Low-Temperature Stress

**DOI:** 10.3389/fcell.2021.635533

**Published:** 2021-01-28

**Authors:** Pan Zhang, Dong Qian, Changxin Luo, Yingzhi Niu, Tian Li, Chengying Li, Yun Xiang, Xinyu Wang, Yue Niu

**Affiliations:** Ministry of Education Key Laboratory of Cell Activities and Stress Adaptations, School of Life Sciences, Lanzhou University, Lanzhou, China

**Keywords:** actin depolymerizing factor, cold stress, C-repeat binding factors genes, actin cytoskeleton, regulatory mechanism

## Abstract

Low temperature is a major adverse environment that affects normal plant growth. Previous reports showed that the actin cytoskeleton plays an important role in the plant response to low-temperature stress, but the regulatory mechanism of the actin cytoskeleton in this process is not clear. C-repeat binding factors (CBFs) are the key molecular switches for plants to adapt to cold stress. However, whether CBFs are involved in the regulation of the actin cytoskeleton has not been reported. We found that *Arabidopsis actin depolymerizing factor 5* (*ADF5*), an ADF that evolved F-actin bundling function, was up-regulated at low temperatures. We also demonstrated that CBFs bound to the *ADF5* promoter directly *in vivo* and *in vitro*. The cold-induced expression of *ADF5* was significantly inhibited in the *cbfs* triple mutant. The freezing resistance of the *adf5* knockout mutant was weaker than that of wild type (WT) with or without cold acclimation. After low-temperature treatment, the actin cytoskeleton of WT was relatively stable, but the actin cytoskeletons of *adf5, cbfs*, and *adf5 cbfs* were disturbed to varying degrees. Compared to WT, the endocytosis rate of the amphiphilic styryl dye FM4-64 in *adf5, cbfs*, and *adf5 cbfs* at low temperature was significantly reduced. In conclusion, CBFs directly combine with the CRT/DRE DNA regulatory element of the *ADF5* promoter after low-temperature stress to transcriptionally activate the expression of *ADF5*; ADF5 further regulates the actin cytoskeleton dynamics to participate in the regulation of plant adaptation to a low-temperature environment.

## Introduction

Low temperature is a major plant stress factor that causes plant growth delay, stagnation, and retrogression and reduces grain yield (Dolferus, 2014; Zhang et al., 2019). Over long evolutionary process, plants have created a series of complex mechanisms to adapt to low-temperature stress. Cold acclimation is one of these mechanisms. When plants are exposed to non-freezing low temperatures for a period of time, their tolerance to lower temperatures improves (Thomashow, 1999; Shi et al., 2018). C-repeat binding factors (CBFs) are the key molecular switches in this process (Liu J. et al., 2018). *CBF1, CBF2*, and *CBF3*, also known as *DREB1B, DREB1C*, and *DREB1A*, belong to the AP2/ERF transcription factor family, which recognizes the C-repeat (CRT)/dehydration responsive element (DRE) DNA regulatory element (CCGAC) (Stockinger et al., 1997; Gilmour et al., 1998; Liu et al., 1998; Medina et al., 1999; Thomashow, 1999). Low temperature rapidly induces the expression of *CBFs*. CBFs further activate the expression of a set of *COLD-REGULATED* (*COR*) genes (Chinnusamy et al., 2007; Ding et al., 2019). These genes generally encode some cryoprotective proteins, reactive oxygen species scavenging proteins, enzymes for osmolyte biosynthesis and photosynthetic membrane protective proteins, which enhance the freezing resistance of plants (Lin and Thomashow, 1992; Jaglo-Ottosen et al., 1998; Liu et al., 1998; Janmohammadi et al., 2015; Bremer et al., 2017).

The maintenance and establishment of specific structures and highly dynamic changes in the plant actin cytoskeleton are necessary for plant cellular processes, such as cell division, cytoplasmic streaming, and vesicular transport (Staiger, 2000). The rapid remodeling of actin cytoskeleton is directly regulated by diverse actin-binding proteins (ABPs), such as profilin, ADF/cofilin, capping protein, villin/gelsolin, formin, and actin-related protein2/3 (Arp2/3) complex (Staiger and Blanchoin, 2006; Qian and Xiang, 2019). Actin depolymerizing factors (ADFs) are conserved actin binding proteins in eukaryotes that regulate the actin cytoskeleton by forming more pointed ends and monomer actins via their severing/depolymerizing activity (Staiger et al., 1997; Hussey et al., 2002; Andrianantoandro and Pollard, 2006). The *Arabidopsis* genome encodes 11 ADF genes, which are divided into 4 subfamilies (Ruzicka et al., 2007). Our laboratory reported that the third subfamily of *Arabidopsis thaliana* (ADF5, ADF9) have developed new functionalization during the process of evolution. They lost the conservative severing/depolymerizing activity of the family and evolved F-actin bundling function (Nan et al., 2017). The other three subfamilies retain the conserved function of severing/depolymerizing activities (Tholl et al., 2011; Nan et al., 2017). The physiological functions of plant ADFs were reported in recent years, such as pollen germination, pollen tube polar growth (Chen et al., 2002, 2003; Bou Daher et al., 2011; Zheng et al., 2013; Jiang et al., 2017; Zhu et al., 2017), hypocotyl elongation (Dong et al., 2001; Henty et al., 2011), stomatal movement (Zhao S. et al., 2016; Qian et al., 2019), innate immunity (Tian et al., 2009; Porter et al., 2012; Fu et al., 2014; Inada et al., 2016), and nematode and aphid infection (Clément et al., 2009; Mondal et al., 2018). The new function of ADF5 plays an important role in regulating the actin bundling process during certain plant-specific physiological activities, such as pollen germination, pollen tube polar growth and ABA-induced stomatal closure (Zhu et al., 2017; Qian et al., 2019). ADF9 is primarily involved in plant growth and development. Under long-day light cycles, the *adf9* mutant showed an early flowering phenotype. Compared to the WT, *adf9* had delayed growth, a reduced number of lateral branches and a weakened callus formation ability (Burgos-Rivera et al., 2008). Previous studies showed that wheat *ADF* (*TaADF*) was rapidly and strongly up-regulated under low temperature (Danyluk et al., 1996). Bioinformatics analysis and previous reports showed that the expression of *ADFs* in *Arabidopsis thaliana* changed under low temperature stress (Fan et al., 2015), which suggests that members of this family also participate in the plant response and adaptation to low-temperature stress. However, the physiological function and molecular mechanisms of ADF members after low-temperature stress are not clear.

We found that CBFs directly bound to the CRT/DRE DNA regulatory element of *the ADF5* promoter to induce the up-regulation of *ADF5* expression and finely regulated actin cytoskeleton dynamics at low temperature and affected the endocytosis rate of the FM4-64-labeled plasma membrane. The present study identified the molecular module of the CBF pathway regulating the actin cytoskeleton at low temperature, which enriched the physiological function of ADF5 and revealed the potential mechanism of the actin cytoskeleton response to cold stress.

## Results

### ADF5 Promotes Basic and Acquired Freezing Resistance in *Arabidopsis thaliana*

**Figure 1:**
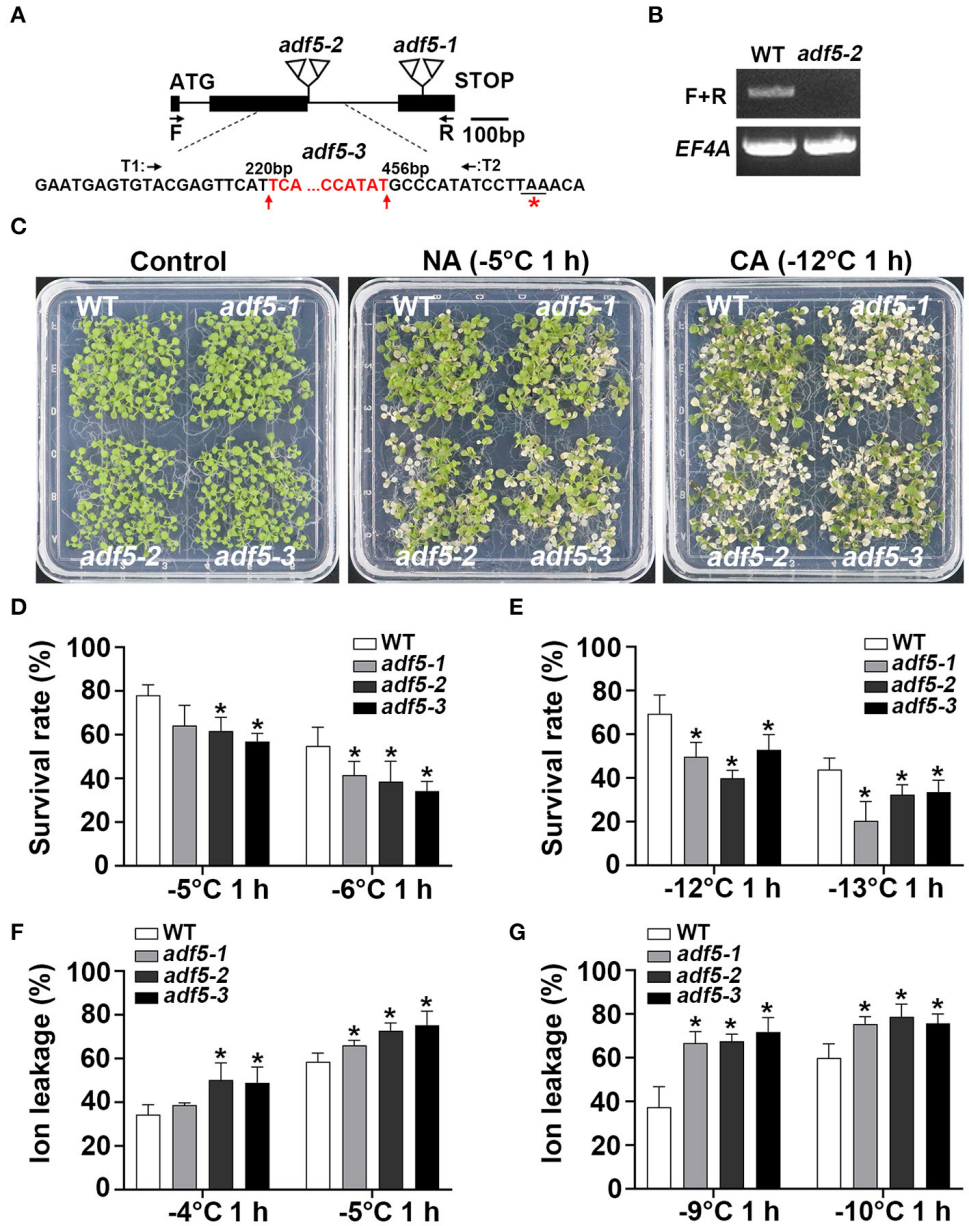
Mutation of *ADF5* results in increased freezing sensitivity. **(A)** Sketch map of *adf5* mutants. The black square represents the exon, and the black line represents the intron. The positions of *adf5-1* and *adf5-2* T-DNA are shown in the triangle. F and R are amplification sites of *ADF5* for RT-PCR or identification of *adf5-3*. T1 and T2 are specific Cas9-splicing sites. The red base is deleted in *adf5-3*, and the red asterisk is the edited termination site. **(B)** Expression of full length *ADF5* in WT and *adf5-2*. *EF4A* is used as an internal control gene. **(C)** Freezing phenotypes of WT and *adf5s*, each material contains ~40 seedlings, which were directly used for freezing treatments (non-cold-acclimated, NA), or treated at 4°C for 3 days (cold-acclimated, CA) before freezing treatment. **(D,E)** Survival rate of WT and *adf5s*. After 3–5 days of recovery under normal growth conditions, the survival rate was calculated. The data are the means of three biological replicates ± SD (*n* = 40 for each replicate). Asterisks indicate statistically significant differences (*P* < 0.05, one-way ANOVA with a Dunnett's multiple comparisons test). **(F,G)** Ion leakage of WT and *adf5s*. After 3 weeks of normal growth in soil, the fully developed rosettes of seedlings were used for freezing treatment to obtain ion leakage under NA and CA conditions. The data are the means of three biological replicates ± SD (*n* = 5 for each replicate). Asterisks indicate statistically significant differences (*P* < 0.05, one-way ANOVA with a Dunnett's multiple comparisons test).

The bioinformatics data showed that low temperature treatment up-regulated the expression of *ADF5* and inhibited *ADF9* expression (Nan et al., 2017), which suggests that this subfamily is involved in the plant response to low-temperature stress. To reveal the physiological function of *Arabidopsis ADF5* under low-temperature stress, we used three *adf5* knockout/gene editing mutants: *adf5-1, adf5-2*, and *adf5-3*. Our laboratory previously reported *adf5-1* (Zhu et al., 2017). The *adf5-2* is a T-DNA insertion mutant (SALK_030145) from ABRC. PCR and sequencing showed that two T-DNAs were inserted after the first G of the second intron (Figure 1A). RT-PCR showed that the full-length *ADF5* gene in *adf5-2* was not expressed (Figure 1B). The *adf5-3* was generated by CRISPR/Cas9 technology in which the 236 bp of *ADF5* genome was deleted (221 to 455 bp after ATG) and only 21 amino acids were correctly translated (Figure 1A).

To verify the phenotype of these mutants under low temperature stress, we first used 12 to 15-day-old seedlings grown on 1/2 MS plates for freezing analyses. The results showed that the three *adf5* knockout mutants had lower freezing resistance and lower survival rate than WT with or without cold acclimation, and the difference was statistically significant (Figures 1C–E). Ion leakage generally represents damage to the cell membrane under stress, and it negatively correlates with the survival rate of plants after freezing (Ye et al., 2019). Second, we used seedlings that grew in soil for 21 days to perform independent freezing treatment experiments and obtained the ion leakage of WT and *adf5* with or without cold acclimation. The ion leakage of *adf5* was higher than WT under both treatment conditions, and the difference was statistically significant (Figures 1F,G). In conclusion, the results of freezing experiments and physiological data indicate that ADF5 promotes basic and acquired freezing resistance in *Arabidopsis thaliana*.

### Low Temperature Can Induce the Expression of *ADF5* Through a Partial CBFs Dependent Pathway

**Figure 2:**
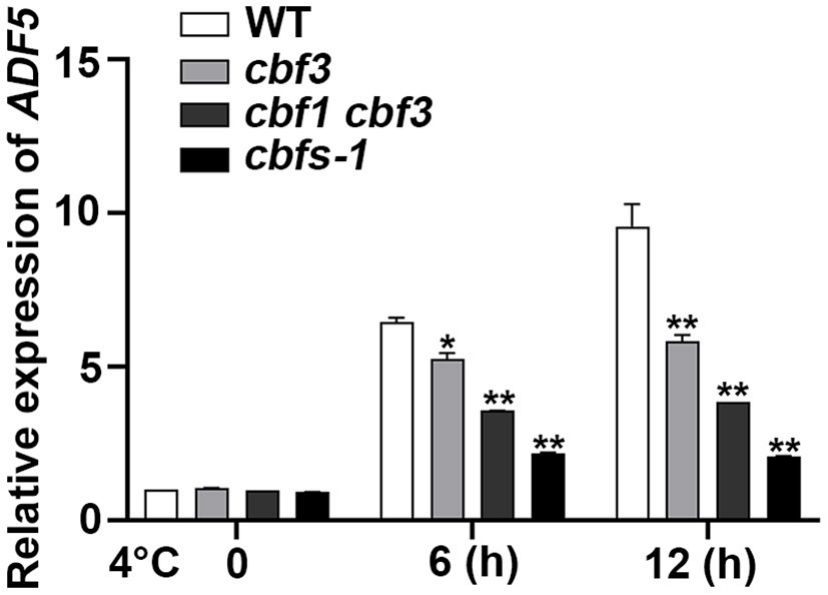
*ADF5* expression is induced by cold in *WT, cbf3, cbf1/3*, and *cbfs*. Ten-day-old *Arabidopsis* seedlings were treated at 4°C for different durations, and the leaves were detached for RNA extraction. *UBQ10* was used as an internal standard. Data presented are the means ± SD of three independent biological replicates (*P < 0.05; **P < 0.01; Student's two-tailed *t*-test).

**Figure 3:**
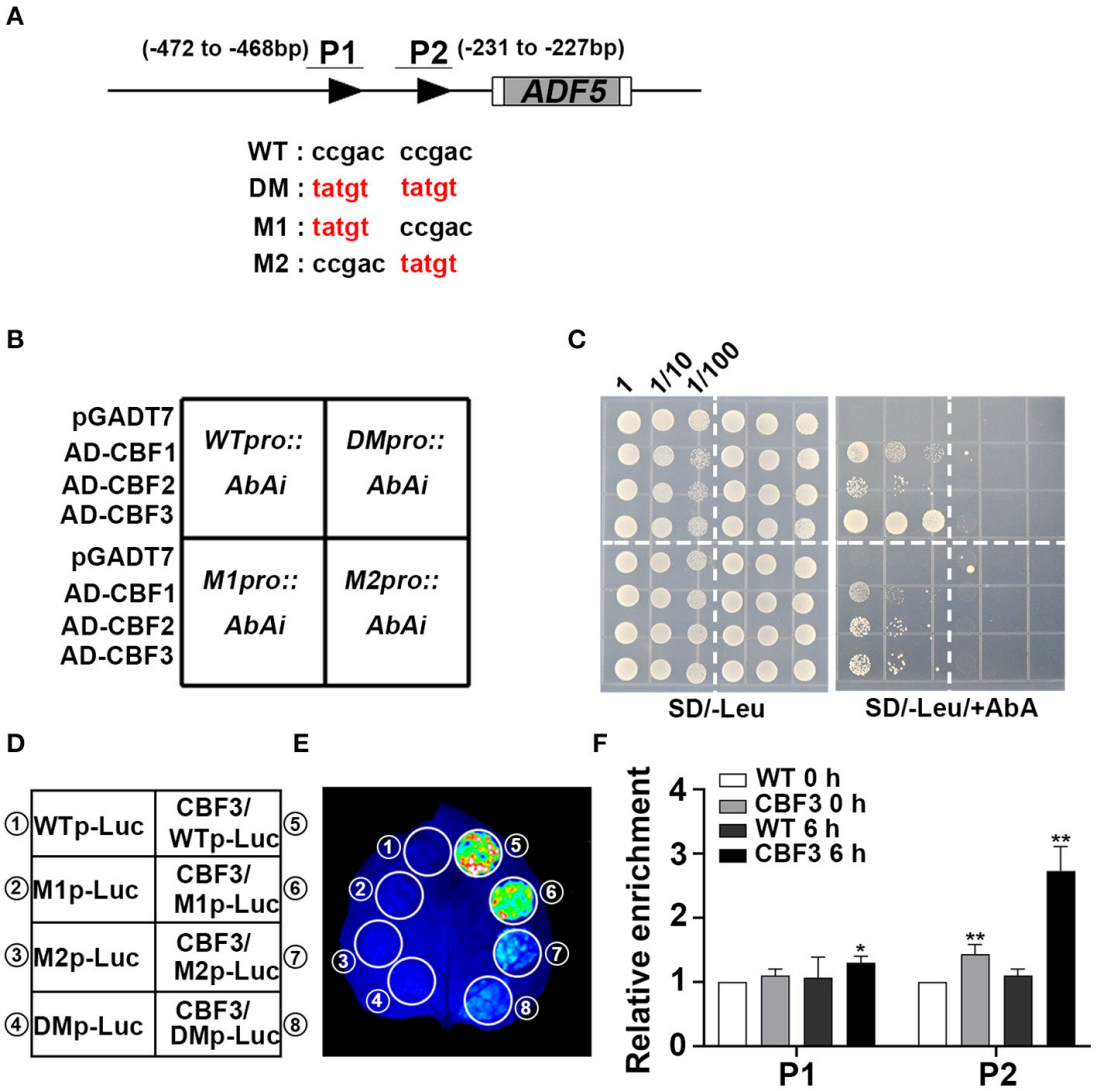
CBFs activate *ADF5* expression via direct binding to the *ADF5* promoter. **(A)** Schematic diagram of the CRT/DRE DNA regulatory element of the *ADF5* promoter. The black triangle represents the CRT/DRE DNA regulatory element. M1 is a mutation at the −472 to −468 bp sites. M2 is a mutation at the −231 to −227 bp sites. DM is a simultaneous mutation of M1 and M2. P1 and P2 are specific primers for ChIP assay. **(B)** Diagram of Y1H colony. **(C)** Y1H assay of the interaction between CBFs and the *ADF5* promoter showing the growth of yeast cells on SD/-Leu medium containing 850 ng/mL Aureobasidin A (AbA). The numbers above the images indicate the dilutions. **(D)** Schematic diagram of the transcriptional activation experiment. **(E)** CBF3 activates the expression of *ADF5* in tobacco leaves via transient expression. **(F)** ChIP analysis of the interaction between CBFs and the *ADF5* promoter under normal conditions or 4°C for 6 h. P1 contains −472 to −468 bp sites of the CRT/DRE DNA regulatory element. P2 contains −231 to −227 bp sites of the CRT/DRE DNA regulatory element. Data presented are the means ± SD of three independent biological replicates (*P < 0.05; **P < 0.01; Student's two-tailed *t*-test).

To verify whether the bioinformatics analysis is correct, we used qRT-PCR to detect the expression of *ADF5* in the WT at low temperature. *ADF5* was up-regulated nearly 10-fold after 12 h at 4°C, which indicates that *ADF5* is a cold-induced gene (Figure 2). To examine the transcriptional regulatory factors upstream of *ADF5*, we analyzed its promoter sequence and found a CCGAC element at −231 to −227 bp and −472 to −468 bp sites, which could be recognized by CBFs (Figure 3A). To demonstrate that CBFs are the upstream transcription activator of *ADF5*, we used the *cbf3* single mutant, *cbf1 cbf3* double mutant and *cbf1 cbf2 cbf3* triple mutant (*cbfs-1*) to detect the induction of *ADF5* at 4°C for different durations. The induction amount of *ADF5* in the single mutant was lower than that in WT, and the double mutant was further reduced. *ADF5* induction was significantly inhibited in the triple mutant (Figure 2). Together, these results indicate that ADF5 may participate in the CBFs signaling pathway.

### *ADF5* Is a Downstream Target Gene of CBFs

To further confirm that CBFs bind to the corresponding region of the *ADF5* promoter, we performed yeast one-hybrid (Y1H) experiment. The results show that CBF1, CBF2, and CBF3 bind to the *ADF5* promoter (Figures 3B,C). We further mutated the CBF recognition sites on the *ADF5* promoter to determine the key binding sites (Stockinger et al., 1997) (Figure 3A). We found the mutation of the two binding sites recognized by CBFs or the binding sites at −231 to −227 bp, CBFs no longer bound to the *ADF5* promoter in yeast, but the sites at −472 to −468 bp remained functional (Figures 3B,C). These results indicate that CBFs bind to the *ADF5* promoter via the CRT/DRE DNA regulatory element at −231 to −227 bp sites.

To determine the effect of CBFs on *ADF5* activation, we performed transient transcriptional activation experiments in tobacco leaves. Because Y1H analysis found that CBF1, CBF2, and CBF3 bound to the *ADF5* promoter, and their function had a certain redundancy (Park et al., 2015), we selected the CBF3 with the strongest binding ability in the Y1H experiments for follow-up experiments (Figures 3B,C). Transcriptional activation analysis showed that CBF3 directly transcriptionally activated the expression of *ADF5* in tobacco mesophyll cells (Figures 3D,E). Subsequent mutation analysis showed that the recognition sites at −472 to −468 bp did not affect the transcriptional activation efficiency of CBF3 to *ADF5* and the mutation at −231 to −227 bp sites or both significantly reduced the transcriptional activation efficiency (Figure 3E). The results indicate that CBF3 activates the expression of *ADF5* via binding to the CBF recognition sites of the *ADF5* promoter at −231 to −227 bp.

To further confirm that CBFs bound to the *ADF5* promoter *in vivo*, ChIP experiments were performed. Col/*pSuper::CBF3-Myc* was treated at 4°C for different times. Chromatin was precipitated using anti-c-myc antibody magnetic beads. P1 primers containing −472 to −468 bp sites and P2 primers containing −231 to −227 bp sites were used to analyze the enrichment by qRT-PCR (Figure 3A). The results showed no significant difference between the *CBF3* overexpression line and the WT at the −472 to −468 bp sites at 0 h, but the *CBF3* overexpression line was 1.3 times more enriched than the WT at the −231 to −227 bp sites. After 6 h of low-temperature treatment, the enrichment of WT at the two recognition sites did not change significantly, and *CBF3* overexpression line showed a significant increase that was nearly three times the enrichment at the −231 to −227 bp sites (Figure 3F). These results suggest that CBF3 binds to the CBF recognition element of the *ADF5* promoter *in vivo*, and this binding activity is regulated by low temperature. In conclusion, CBFs primarily bind to the CBF recognition sites at the −231 to −227 bp of the *ADF5* promoter *in vivo*, and *ADF5* is a downstream target gene of CBFs.

### The Effect of ADF5 on Freezing Resistance in *Arabidopsis thaliana* Partially Depends on the CBF Pathway

**Figure 4:**
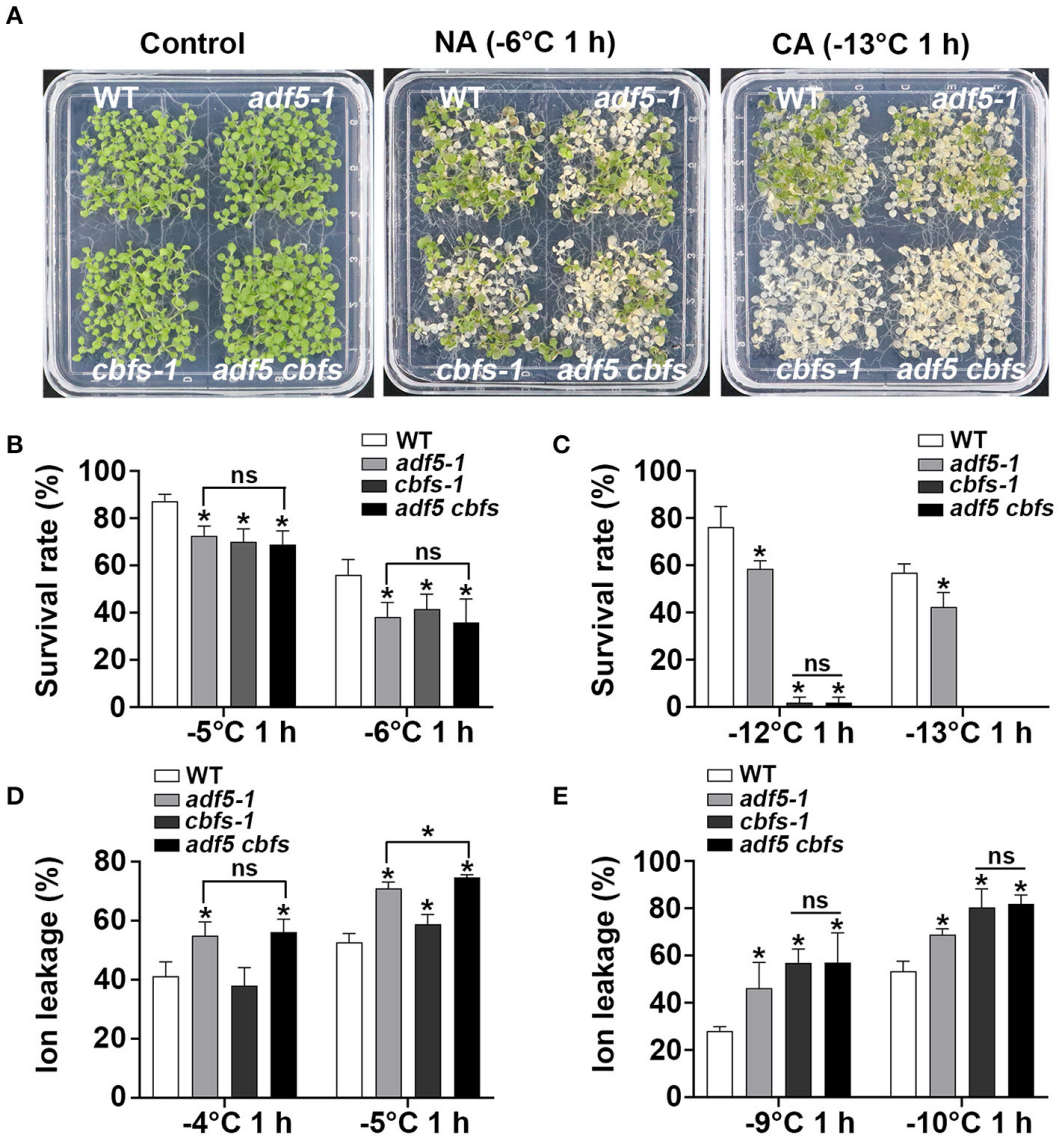
ADF5 is the genetic downstream of CBFs. **(A)** Freezing phenotypes of WT, *adf5-1, cbfs-1*, and *adf5 cbfs* under NA or CA conditions. **(B,C)** Survival rate of WT, *adf5-1, cbfs-1*, and *adf5 cbfs*. After 3–5 days of recovery under normal growth conditions, the survival rate was calculated. The data are the means of three biological replicates ± SD (*n* = 40 for each replicate). Asterisks indicate statistically significant differences and ns is not significant (*P* < 0.05, one-way ANOVA with a Tukey's multiple comparisons test). **(D,E)** Ion leakage of WT, *adf5-1, cbfs-1*, and *adf5 cbfs*. After 3 weeks of normal growth in soil, the fully developed rosettes of seedlings were used for freezing treatment to obtain ion leakage under NA or CA conditions. The data are the means of three biological replicates ±SD (*n* = 5 for each replicate). Asterisks indicate statistically significant differences and ns is not significant (*P* < 0.05, one-way ANOVA with a Tukey's multiple comparisons test).

To further investigate the genetic relationship between ADF5 and CBFs, *adf5 cbfs* quadruple mutant was obtained by crossing *adf5-1* with the triple mutant *cbfs-1*. Freezing experiments showed that *adf5-1, cbfs-1*, and *adf5 cbfs* were more sensitive than WT under non-cold-acclimated (NA) conditions, and the survival rate was significantly different. The freezing resistance of *adf5 cbfs* was closer to *adf5-1* with decreasing temperature and there was no significant difference between the two groups (Figures 4A,B). Under cold-acclimated (CA) conditions, *adf5-1, cbfs-1*, and *adf5 cbfs* were more sensitive to freezing than WT, and their survival rate was significantly different. The freezing resistance of the *adf5 cbfs* showed the freezing resistance of *cbfs-1* and there was no significant difference between the two groups (Figures 4A,C). Seedlings grown in soil for 21 days were used for freezing treatment to obtain ion leakage. Under NA conditions, the ion leakage of WT and *cbfs-1* treated at −4°C was similar, and there was no significant difference. *adf5-1* and *adf5 cbfs* had higher ion leakage than WT and were significantly different. At −5°C, the ion leakage of *cbfs-1* increased significantly relative to WT. The *adf5-1* and *adf5 cbfs* had significantly higher ion leakage than WT, and the *adf5 cbfs* had significantly higher ion leakage than *adf5-1* (Figure 4D). Under CA conditions, the ion leakage of *adf5-1, cbfs-1*, and *adf5 cbfs* was significantly higher than WT. The ion leakage of *adf5 cbfs* resembled *cbfs-1*, and there was no significant difference (Figure 4E). In conclusion, ADF5 positively regulates the freezing tolerance ability of *Arabidopsis thaliana* by partially relying on the CBF pathway.

### The Actin Cytoskeleton of the Mutant Was Disordered During Cold Acclimation

**Figure 5:**
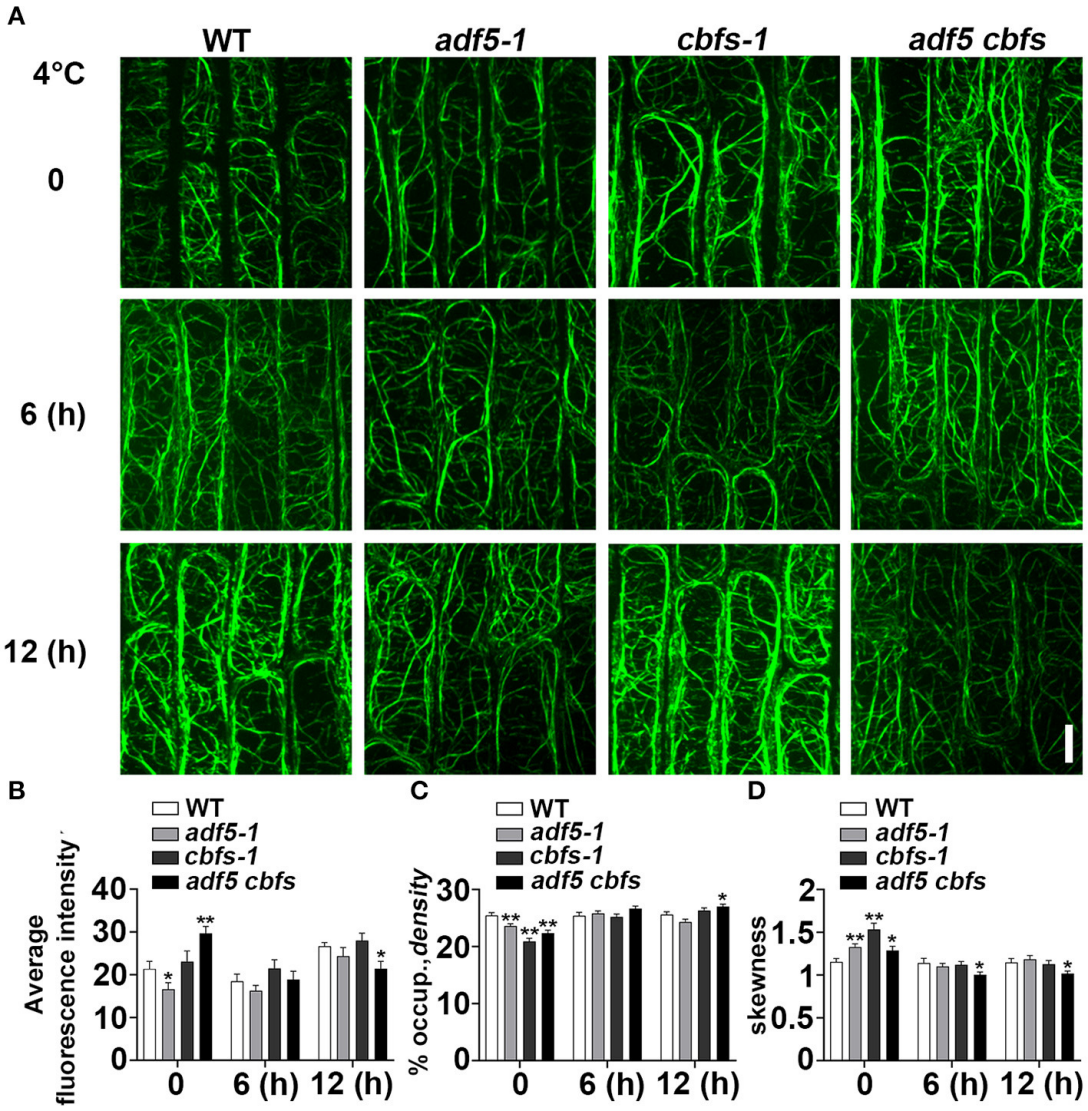
Actin cytoskeleton array rearranges in response to cold treatment. **(A)** Five-day-old seedlings expressing ABD2-GFP were used for fluorescence collection. Images of the cortical actin cytoskeleton array in epidermal cells from the root elongation transition zone of WT, *adf5-1, cbfs-1*, and *adf5 cbfs* treated at 4°C for different durations. Scale bar = 10 μm. **(B)** The average intensity of fluorescence of the GFP signal in WT, *adf5-1, cbfs-1*, and *adf5 cbfs* root elongation transition zone cells. **(C)** Average actin cytoskeleton density of WT, *adf5-1, cbfs-1*, and *adf5 cbfs* root elongation transition zone cells. **(D)** The extent of actin cytoskeleton bundling (skewness) of WT, *adf5-1, cbfs-1*, and *adf5 cbfs* root elongation transition zone cells. The values represent the means ± SEM (*n* = 60 cells per genotype. *P < 0.05; **P < 0.01; Student's two-tailed *t*-test).

To determine whether the phenotypes of the *adf5* and *cbfs* mutants are directly related to the actin cytoskeleton, we observed the actin cytoskeleton morphology in epidermal cells from root transition and elongation zone of WT and mutants under normal growth and different durations of 4°C treatment (Figure 5A). ABD2-GFP was expressed in all mutants after crossing Col/*pUBQ10::ABD2-GFP*. The average fluorescence intensity was measured directly. Similar to previous reports, the fluorescence intensity of *adf5-1* under normal growth conditions was significantly lower than the WT (Zhu et al., 2017; Qian et al., 2019). The fluorescence intensity of *cbfs-1* was slightly higher than WT, but *adf5 cbfs* had the highest fluorescence intensity compared to WT, with a significant difference. After 6 h of low temperature treatment, the fluorescence intensity of WT and *cbfs-1* decreased, and *adf5 cbfs* obviously decreased, but the fluorescence intensity of *adf5-1* was not changed. There was no significant difference among the materials at this time. After 12 h of low-temperature treatment, the fluorescence values of all materials recovered, and there was no significant difference among WT, *adf5-1*, and *cbfs-1*. The fluorescence value of the *adf5 cbfs* quadruple mutant increased less, and there was a significant difference between WT and *adf5 cbfs* (Figure 5B).

We analyzed the actin cytoskeleton density and skewness after different time points of low-temperature treatment (Figures 5C,D). Consistent with a previous report, the structure of the actin cytoskeleton in WT was relatively stable (Shibasaki et al., 2009), and its density and degree of filament bundling (skewness) were not changed. Compared to WT, the mutants had lower actin cytoskeleton density and a higher degree of bundled actin filament at 0 h, which corresponded to the function of ADF5 in forming a more stable actin cytoskeleton network (Zhu et al., 2017; Qian et al., 2019). After 6 hours of low-temperature treatment, the actin cytoskeleton density in the *adf5-1, cbfs-1*, and *adf5 cbfs* mutants was relatively changed. However, there was no significant difference between WT and mutants. The degree of bundled actin filament was reduced, there was no significant differences among the *adf5-1, cbfs-1*, and WT, but significant differences between *adf5 cbfs* and WT. After 12 h of treatment, the density of *adf5-1* decreased slightly and *cbfs-1* increased, but there was no significant difference compared to WT. The quadruple mutant also increased and was significantly different than WT. The skewness value of *adf5-1* increased, and the values of *cbfs-1* and *adf5 cbfs* decreased. Only the quadruple mutant was significantly different from WT (Figures 5C,D). In conclusion, the actin cytoskeleton structure of WT is relatively stable, but the mutants change significantly compared to WT under low temperature treatment, and actin cytoskeleton dynamic process in mutants is affected.

### The Endocytosis Rate of the Mutants Decreased During Cold Acclimation

**Figure 6:**
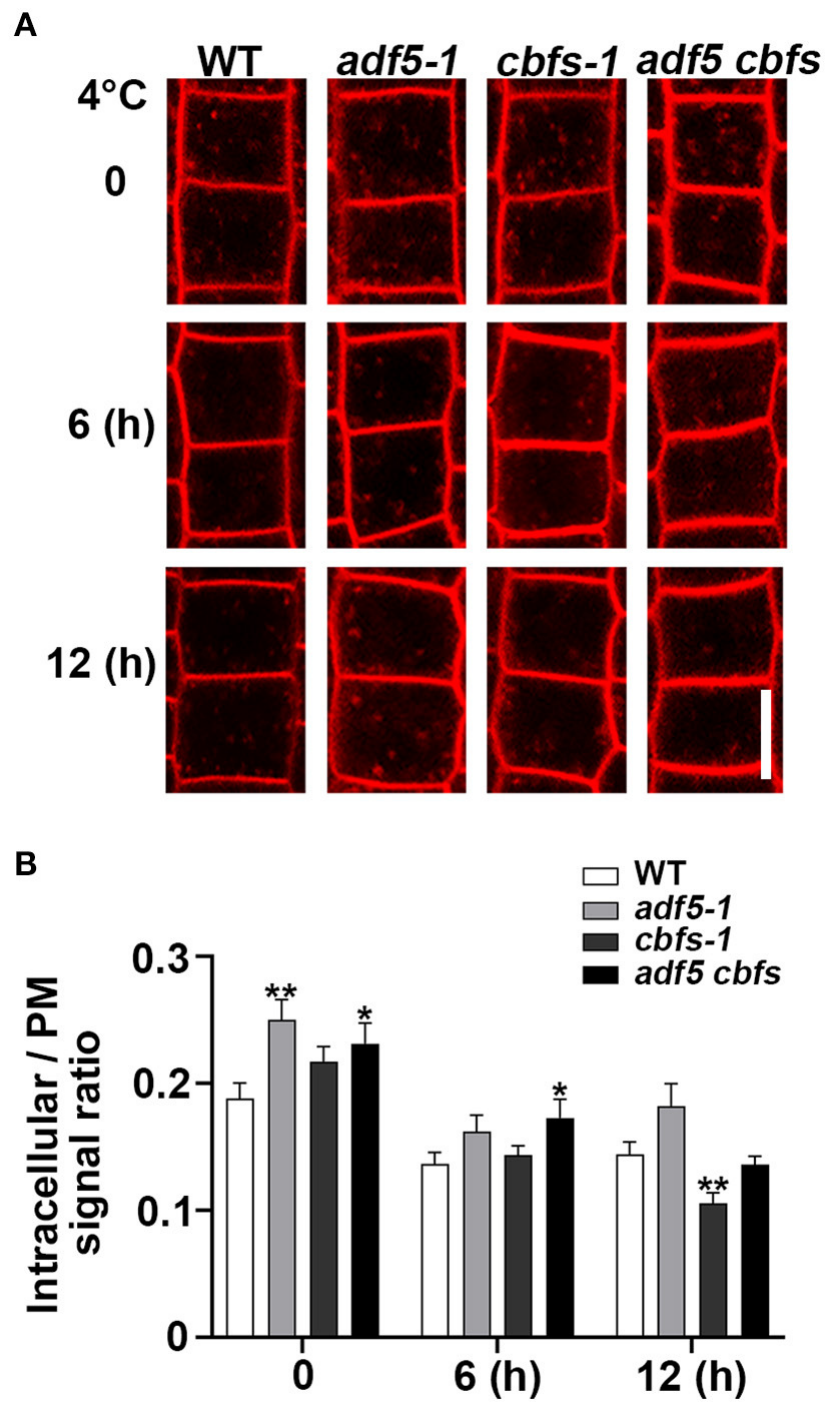
Low temperature reduced the rate of endocytosis in mutants compared to WT. **(A)** Five-day-old seedlings were used for amphiphilic styryl dye FM4-64 signal intensity collection. Seedling roots of WT, *adf5-1, cbfs-1*, and *adf5 cbfs* treated at 4°C for different durations were labeled with FM4-64 to measure internalization. Scale bar = 10 μm. **(B)** The rate of cytosol average fluorescence intensity to plasma membrane average fluorescence intensity. The values represent the means ± SEM (*n* = 60 cells per genotype. *P < 0.05; **P < 0.01; Student's two-tailed *t*-test).

The dynamics of the plasma membrane are very important for the plant response to stress, and the actin cytoskeleton is important for plant endocytosis (Baluška et al., 2002; Wang et al., 2020). Considering the effect of low temperature on actin cytoskeleton dynamics, then to track the endocytosis process, we used amphiphilic styryl dye FM4-64 to label epidermal cells from root transition zone and quantified the cytosol/PM FM4-64 signal intensity ratio to represent the endocytosis rate (Figures 6A,B). The results showed that the endocytosis rate of *adf5-1, cbfs-1*, and *adf5 cbfs* mutants was faster than WT, and there was significant differences between *adf5-1, adf5 cbfs*, and WT, respectively, but there was no significant difference in *cbfs-1*. The endocytosis rate of WT, *adf5-1, cbfs-1*, and *adf5 cbfs* decreased after 6 h of low-temperature treatment, and the difference between WT and mutants was reduced. There was a significant difference between *adf5 cbfs* and WT. After 12 h of low-temperature treatment, the endocytosis rate of WT and *adf5-1* increased slightly, and there was no significant difference between *adf5* and WT. The endocytosis rate of *cbfs-1* and *adf5 cbfs* was further reduced, and there was a significant difference between *cbfs-1* and WT, but not in the quadruple mutant (Figure 6B). The results indicate that the abnormal endocytosis rate of *adf5-1, cbfs-1*, and *adf5 cbfs* under normal growth conditions may be the reason for their freezing sensitivity. During the cold training process, the endocytosis rate of non-synchronous change in mutants may cause disorders of cold signals and cryoprotective substance transport, which may affect the freezing resistance of the mutants.

## Discussion

Previous reports showed that low temperature disturbed the actin cytoskeleton of plant cells and reduced all cell processes (Das et al., 1966; Pokorná et al., 2004; Fan et al., 2015). The present study provided molecular genetic and physiological evidences for ADF5, which is an ABP that evolved F-actin-bundling function, regulation of actin cytoskeleton dynamics at low temperature partially via a CBF-dependent pathway. The molecular components of fine regulation of the actin cytoskeleton at low temperature were demonstrated.

Cold acclimation reshapes the physiological and biochemical status of cells (Janmohammadi et al., 2015; Ding et al., 2019). CBFs are the core regulatory factor in this process and activate many downstream *COR* genes during cold acclimation (Shi et al., 2018). Published transcriptome data revealed that CBFs regulated a number of potential actin cytoskeleton regulatory genes including *ADF5* (Jia et al., 2016). Previous evidence also showed that TaADF accumulated more in freezing-resistant seedlings (Ouellet et al., 2001). Our qRT-PCR data also directly indicated that *ADF5* was partially up-regulated by CBFs at low temperature treatment (Figure 2). Genetic analyses also showed that the freezing sensitivity of *adf5 cbfs* was closer to *adf5-1* under NA conditions (Figure 4A). Under this condition, the ion leakage of *adf5 cbfs* was higher than *adf5-1*, and the difference was statistically significant (Figure 4D). These results suggested that ADF5 promotes the freezing resistance of *Arabidopsis thaliana* via a non-CBF pathway. Under CA conditions, CBFs are the core transcription factors of the cold response pathway, and there are many downstream induced genes. The *adf5 cbfs* primarily showed the freezing sensitivity and physiological characteristics of *cbfs-1* (Figures 4A,E). In summary, ADF5 promotes freezing resistance in *Arabidopsis thaliana* partially via the CBF signaling pathway. Moreover, transcriptome data clearly showed that some non-CBF-dependent ABPs were cold regulated (Jia et al., 2016; Zhao C. et al., 2016). For example, *AtFH16* was downregulated, and *FIM4* and *CROLIN* were up-regulated (Jia et al., 2013; Wang J. et al., 2013; Ding et al., 2018). Therefore, coordination of actin cytoskeleton dynamics is an important plant cell process under low-temperature stress.

Membrane vesicle transport is a conserved basic cell process in eukaryotes, and it plays an important role in the plant response to stress. Under stress, it is responsible for the correct sorting and positioning of specific proteins and the stress response (Rosquete and Drakakaki, 2018; Wang et al., 2020). For example, low temperature reduces the intracellular transport of auxin transporters PIN2 and PIN3, which affects auxin transport and changes the gravitropism of roots (Shibasaki et al., 2009). Recent studies also showed that stable GNOM ARF-GEF-mediated endosomal trafficking helped *Arabidopsis* adapt to low temperature (Ashraf and Rahman, 2019). The cryoprotective protein RCl2A also depends on clathrin-mediated endocytosis (Wang C. et al., 2013), and the transport of its homologous protein RCI2B at low temperature is selectively regulated to maintain a normal transport rate (Shibasaki et al., 2009). The actin cytoskeleton plays an important role in endocytosis and intracellular transport (Šamaj et al., 2004; Kim et al., 2005). Previous studies showed that BRI1 and CRPK1 were essential for the regulation of plant freezing resistance (Liu et al., 2017; Unterholzner et al., 2017). BRI1 is internalized by clathrin and sorted by the SYP61/VHA-a1 endosomal compartment, which also sorts the auxin transporters PIN2 and AUX1(Robert et al., 2008). This process requires coordination of the actin cytoskeleton (Lanza et al., 2012; Arieti and Staiger, 2020). The number of actin cytoskeletons decreased in the rice *vln2* mutant, and the polarity distribution and cycle of PIN2 changed (Wu et al., 2015). FH5-regulated actin cytoskeleton polymerization and elongation processes are necessary for the movement of FH5-labeled vesicles in *Arabidopsis* pollen tubes (Liu C. et al., 2018). Therefore, the actin cytoskeleton is very important for the integrity of the endocytosis and sorting processes, which ultimately affects the correct positioning and distribution of endocytic cargos and the plant response to stress. However, the processes of vesicle transport and sorting controlled by the actin cytoskeleton under low temperature are rarely reported.

The present study showed that the morphology of the actin cytoskeleton in WT was relatively stable consistent with previous reports (Shibasaki et al., 2009; Figure 5), and its endocytosis rate was reduced during low-temperature treatment (Figure 6), which may be related to the slowing of molecular thermal movement at low temperature. However, the endocytosis rate in *adf5-1* had a greater reduction than WT at 6 h, which may be related to the disorder of the *adf5-1* actin cytoskeleton (Figures 5, 6). The endocytosis rate of *cbfs-1* and *adf5 cbfs* continuously decreased significantly compared to WT at low temperature (Figure 6), which may be due to the influence of their disturbed actin cytoskeleton and additional regulatory pathways regulated downstream of CBFs. In summary, the damaged actin cytoskeleton of mutants changes the endocytosis rate during cold acclimation and the change in the endocytosis rate is not consistent with WT, in which the distribution, recovery and sorting of receptors and cryoprotective substances on the plasma membrane are directly changed. Therefore, the cold acclimation process of mutants is affected, and the freezing resistance of mutants is reduced.

## Methods

### Plant Materials and Growth Conditions

*Arabidopsis* seeds were vernalized for 3 days at 4°C with 2% PPM-Preservative (Plant Cell Technology) and grown on half-strength Murashige and Skoog medium (MS, PhytoTech M519) containing 0.8% agar and 1.5% sucrose for 12–15 days at 22°C with an optical density of 80–100 μmol m^−2^ s^−1^ under a 16 h light/8 h dark LD photoperiod. *Arabidopsis thaliana* Col-0 and *Nicotiana benthamiana* were used in this experiment. Mutants *adf5-1* (Salk_018325) and *adf5-2* (SALK_030145) were obtained from the *Arabidopsis* Biological Resource Center. *adf5-3* was obtained using two specific Cas9-cleaved targets of *ADF5* to mutate Col-0, and this method was obtained from the report (Xing et al., 2014). The *cbf3, cbf1 cbf3, cbfs-1*, and Col/*pSuper::CBF3-Myc* were obtained from the Prof. Shuhua Yang Laboratory (China Agricultural University) (Jia et al., 2016; Liu et al., 2017). The *adf5 cbfs* was obtained by crossing *adf5-1* with *cbfs-1*. The primers used to identify homozygous lines and for CRISPR are listed in Supplementary Table 1. The materials for ion leakage measurements were grown on MS plates containing 1% agar for 5–7 days, transferred to soil, then grown at 23°C, 16 h light/8 h dark LD photoperiod to an optical density of ~100 μmol m^−2^ s^−1^ and relative humidity of 60% for 3 weeks. *Nicotiana benthamiana* had similar growth conditions.

### Freezing Tolerance and Ion Leakage Assays

This experiment was performed according to the protocol described previously (Shi et al., 2012). Briefly, 12 to 15-day-old seedlings were used to obtain phenotypes by freezing treatment. For cold acclimation (CA) treatment, the normally growing seedlings were grown at 4°C for an additional 3 days at a light density of ~25 μmol m^−2^ s^−1^, 16 h light/ 8 h dark LD photoperiod and transferred to the freezing incubator (PERCIVAL, LT-36VLC8) for treatment according to the following procedure. The seedlings were kept at 0°C for 1 h, then decreased by 1°C per hour until the temperature shown in the figure was maintained for the corresponding times. Non-cold-acclimation (NA) processing was directly performed according to a program. After freezing treatment, the seedlings were shifted to 4°C, kept in darkness for 12 h, and transferred to a normal growth environment for growth recovery for 3–5 days. The survival rate was obtained by calculating the proportion of seedlings that grew new leaves.

Ion leakage assays was performed as described (Guo et al., 2002). Briefly, seedlings grown for 3 weeks in soil were treated for ion leakage analyses with or without cold acclimation (4°C for 3 d). A fully and well-developed rosette leaf was washed with deionized water, and placed in the bottom of a 15-mL sterile centrifuge tube containing 100 μL deionized water, then placed in a low-temperature circulator (SCIENTZ, DC-2030). After holding at 0°C for 30 min, a small amount of tiny and pure ice crystal was added to the centrifuge tube, and the temperature was reduced by 1°C per 30 min until the temperature shown in the figure and maintained for 1 h. The centrifuge tube was removed and kept in darkness at 4°C for 12 h. Then, 10 mL of deionized water was added to the sterile tube, and the tubes were shaken at normal temperature for 2 h. The electrical conductivity S1 was measured. After sterilization at 121°C for 15 min, S2 conductivity was measured after shaking for 2 h. The S0 conductivity of deionized water used in the experiment, and the conductivity S0′ after sterilization was measured. The ratio of S1–S0 to S2–S0′ was used as the ion leakage.

### RNA Extraction and Real-Time Quantitative PCR Analysis

Seedlings grown in a normal environment for 10 days on MS plates containing 1% agar were used for gene expression analyses with or without treatment at 4°C. Total RNA was extracted using a MiniBEST Plant RNA Extraction kit (TaKaRa), and total RNA was reverse transcribed into cDNA using M-MLV reverse transcriptase (TaKaRa). qRT-PCR was performed using SYBR Premix Ex Taq (TaKaRa), and *UBQ10* was used as an internal reference gene. The primers used for qRT-PCR are listed in Supplementary Table 1.

### Yeast One-Hybrid Assays

Yeast one-hybrid (Y1H) assays were performed as described previously (Qian et al., 2019). Briefly, the *ADF5* promoter containing the CRT/DRE DNA regulatory element was amplified using PCR and cloned into the pAbAi vector. The CRT/DRE mutation sequence was obtained using specific primers and cloned into the pAbAi vector. The vector was linearized and transformed into the Y1H GOLD (Clontech) strain. The full-length *CBF* coding sequence was amplified using PCR, cloned into the pGADT7 vector, and transformed into the constructed Y1H strain. Aureobasidin A (AbA, 850 ng/mL, Clontech) was used for the reporter gene activation test. The primers used to clone are listed in Supplementary Table 1.

### Transcriptional Activation Assays

This experiment was performed as described previously (Qian et al., 2019). PCR amplified and cloned the *ADF5* promoter sequence into pGWB235-LUC to construct the reporter vector. The full-length *CBF3* coding sequence was amplified using PCR, and it was cloned into the pBIB-35s-GWR-flag vector as the effect vector. The reporter and effector vectors were co-transformed into *N. benthamiana* leaves for transcriptional activation analyses. A reporting vector alone was used as a negative control. Fluorescence of the luciferase and luciferin (Promega) reaction was obtained using a Lumazone CA1300B camera (Photometrics). The primers used to clone are listed in Supplementary Table 1.

### Chromatin Immunoprecipitation Assays

Under normal growth conditions, seedlings grown on 1% agar MS plates for 12 days were used in this experiment. The seedlings of Col/*pSuper::CBF3-Myc* and Col-0 (without Myc-tag, as negative control) were treated at 4°C or not, and a ChIP experiment was performed according to a previously described method (Ni et al., 2009). The relative enrichment of the *ADF5* promoter region was detected by qRT-PCR using specific primers. The specific primers are listed in Supplementary Table 1.

### Visualization of Actin Filaments by Confocal Laser Scanning Microscopy and Quantitative Analyses of Actin Filament

To visualize the actin cytoskeleton, we crossed Col-0/*pUBQ10::ABD2-GFP* (Tian et al., 2015) with *adf5-1, cbfs-1*, and *adf5 cbfs* and expressed ABD2-GFP in mutants for GFP fluorescence collection. Under normal growth conditions, seedlings grown on 1% agar MS plates for 5 days were treated with or without 4°C for the duration shown in the figure for fluorescence acquisition. Images of epidermal cells from root transition and elongation zone were acquired using a spinning disk microscope (Andor) equipped with a 63 × NA oil immersion lens, and the Z-series images were captured with the step size set at 0.5 μm (Zhou et al., 2020). GFP was excited with a 488-nm laser and observed using a 514-nm emission filter. Images were processed and analyzed using ImageJ. To measure the extra actin cytoskeleton structure, the percentage of occupancy (density) and skewness were measured using ImageJ software as previously described (Higaki et al., 2010; Henty et al., 2011).

### FM4-64 Labeling and Endocytosis Measurements

Under normal growth conditions, the seedlings growing on 1% agar MS plates for 5 days were treated with or without 4°C for different times then used in this experiment. Seedlings were held on ice for 2 min with 2 μM FM4-64 and stained at 22 or 4°C for an additional 5 min. After FM4-64 washout, dye internalization was imaged using a Zeiss LSM880 confocal microscope equipped with 40 × 1.3 NA oil immersion lens. FM4-64 signals were excited with a 488-nm laser and observed using a 600 to 630-nm emission filter. The endocytosis rate was measured and calculated in ImageJ software as previously described (Zhang et al., 2020).

## Data Availability Statement

The raw data supporting the conclusions of this article will be made available by the authors, without undue reservation.

## Author Contributions

YuN conceived the study and designed the research. PZ, DQ, CL, YiN, TL, and CL performed the research. PZ, DQ, CL, YX, XW, and YiN analyzed the data. PZ, YuN, YX, and DQ wrote the manuscript.

## Conflict of Interest

The authors declare that the research was conducted in the absence of any commercial or financial relationships that could be construed as a potential conflict of interest.
