# Supplementary material for: Arabidopsis ADF5 Acts as a Downstream Target Gene of CBFs in Response to Low-Temperature Stress
Source: Front Cell Dev Biol. 2021 Jan 28;9:635533. doi: 10.3389/fcell.2021.635533 (PMC7876393; doi:10.3389/fcell.2021.635533)
Supplement: Supplementary file 1 [file Data_Sheet_1.PDF]

Primers for homozygote identification

Primer name	Primer Sequence
F	ATGGCGATGGCTTTCAAGATG
R	CTATTTGGCACGGTCTTGGA
EF4A-F	TTGGCGGCACCCTTAGCTGGATCA
EF4A-R	ATGCCCCAGGACATCGTGATTTCAT
LBb1.3	ATTTTGCCGATTTCGGAAC
cbf3-LP	TCCATAACGATACGTCGTCATC
cbf3-RP	AGTCTTCTCTGGACACATGGC
LB1	GCCTTTTCAGAAATGGATAAATAGCCTTGCTTCC

Primers for Y1H assays

Primer name	Primer Sequence
ADF5p-Y1H-F	AAGCTTTTGATTTTCTTTGTAATCCC
ADF5p-Y1H-R	GGTACCATCACTATTTACTCTTATTAC
M1-F	GTGAAGAGAGTGTATGTGAGAATGTTTTTC
M1-R	GAAAAACATTCTCACATACACTCTCTTCAC
M2-F	GTGACGTTAATATGTATGTGAGAAAAAGAA
M2-R	TTCTTTTTCTCACATACATATTAACGTCAC
CBF1(UTR)-F	AGAGAGAGAGATATAAATAGCTTTACC
CBF1(UTR)-R	TGGAAACGACTATCGAATATTAGT
CBF1-AD-F	GAATTCATGAACTCATTTTCAGCTTTTTCTG
CBF1-AD-R	GGATCCTTAGTAACTCCAAAGCGACACG
CBF2(UTR)-F	CCTGAATTAGAAAAGAAAGAT
CBF2(UTR)-R	AAAGATTATATATTCTGC
CBF2-AD-F	GAATTCATGAACTCATTTTCTGCCTTTTCTG
CBF2-AD-R	GGATCCTTAATAGCTCCATAAGGACACGTCA
CBF3(UTR)-F	CCTGAACTAGAACAGAAAGAG
CBF3(UTR)-R	CTGAAACTGAATCAATTT
CBF3-AD-F	GAATTCATGAACTCATTTTCTGCTTTTTCTG
CBF3-AD-R	GGATCCTTAATAACTCCATAACGATACGTCG

Primers for RT-qPCR and ChIP PCR

Primer name	Primer Sequence
ADF5-F	CGTTTGTGTTTTGATTGTGTTGTTAA
ADF5-R	CCGTTACTCGTAGGACAAATTCG
UBQ10-F	CACACTCCACTTGGTCTTGCGT
UBQ10-R	TGGTCTTTCCGGTGAGACTCTTCA
P1-F	AAGTGGTGAAGAGAGTGCCGAC
P1-R	GCGTATGGAATTTGGATCA
P2-F	TGCAGAACACACGGTGACG
P2-R	AAAGTTATGCCTATACAAATG

Primers for transient transactivation assays

Primer name	Primer Sequence
ADF5p(GWR)-F	GGGGACAAGTTTGTACAAAAAAGCAGGCTTCTTGATTTTCTTTGTAATCCC
ADF5p(GWR)-R	GGGGACCACTTTGTACAAGAAAGCTGGGTCATCACTATTTACTCTTATTAC
CBF3(GWR)-F	GGGGACAAGTTTGTACAAAAAAGCAGGCTTCATGAACTCATTTTCTGCTTTTTCTG
CBF3(GWR)-R	GGGGACCACTTTGTACAAGAAAGCTGGGTCTTAATAACTCCATAACGATACGTCG

Primers for constructs to generate *adf5-3* mutant by CRISPR/Cas9

Primer name	Primer Sequence
T1-F	ATATATGGTCTCGATTGTGAGTGTACGAGTTCATTCAGTTTTAGAGCTAGAAATAG
T2-R	ATTATTGGTCTCGAAACTATGCCCATATCCTTAAACACAATCTCTTAGTCGACTCTAC
